# Endoscopic versus stereotactic biopsies of intracranial lesions involving the ventricles

**DOI:** 10.1007/s10143-020-01371-7

**Published:** 2020-08-21

**Authors:** Marcin Birski, Jacek Furtak, Kamil Krystkiewicz, Julita Birska, Karolina Zielinska, Paweł Sokal, Marcin Rusinek, Dariusz Paczkowski, Lukasz Szylberg, Marek Harat

**Affiliations:** 1 Neurosurgery Department, 10th Military Research Hospital, ul. Powstancow Warszawy 5, 85-681 Bydgoszcz, Poland; 2 Department of Neurosurgery and Neurology, Jan Biziel University Hospital Collegium Medicum Nicolaus Copernicus University, Bydgoszcz, Poland; 3 Department of Clinical Pathomorphology, Collegium Medicum Nicolaus Copernicus University, Bydgoszcz, Poland; 4 Department of Tumor Pathology and Pathomorphology, Oncology Center, Bydgoszcz, Poland; 5 Department of Pathomorphology, 10th Military Research Hospital, Bydgoszcz, Poland

**Keywords:** Biopsy, Brain tumors, Hydrocephalus, Neuroendoscopy, Stereotactic techniques

## Abstract

Stereotactic biopsies of ventricular lesions may be less safe and less accurate than biopsies of superficial lesions. Accordingly, endoscopic biopsies have been increasingly used for these lesions. Except for pineal tumors, the literature lacks clear, reliable comparisons of these two methods. All 1581 adults undergoing brain tumor biopsy from 2007 to 2018 were retrospectively assessed. We selected 119 patients with intraventricular or paraventricular lesions considered suitable for both stereotactic and endoscopic biopsies. A total of 85 stereotactic and 38 endoscopic biopsies were performed. Extra procedures, including endoscopic third ventriculostomy and tumor cyst aspiration, were performed simultaneously in 5 stereotactic and 35 endoscopic cases. In 9 cases (5 stereotactic, 4 endoscopic), the biopsies were nondiagnostic (samples were nondiagnostic or the results differed from those obtained from the resected lesions). Three people died: 2 (1 stereotactic, 1 endoscopic) from delayed intraventricular bleeding and 1 (stereotactic) from brain edema. No permanent morbidity occurred. In 6 cases (all stereotactic), additional surgery was required for hydrocephalus within the first month postbiopsy. Rates of nondiagnostic biopsies, serious complications, and additional operations were not significantly different between groups. Mortality was higher after biopsy of lesions involving the ventricles, compared with intracranial lesions in any location (2.4% vs 0.3%, *p* = 0.016). Rates of nondiagnostic biopsies and complications were similar after endoscopic or stereotactic biopsies. Ventricular area biopsies were associated with higher mortality than biopsies in any brain area.

## Introduction

Biopsies of brain tumors involving the ventricular system require careful planning. Tissue samples from this area can be obtained using stereotactic or endoscopic methods. With stereotactic biopsies, the long trajectory and potential need to traverse the ependyma may increase the risk of intraventricular bleeding and reduce accuracy. Because of this, endoscopic biopsies have become increasingly used. During endoscopic procedures, the site of tissue collection and any areas of bleeding are continuously visualized. Furthermore, endoscopic procedures also allow simultaneous treatment of hydrocephalus, in addition to tissue sampling.

At the end of the twentieth century, stereotactic biopsy was recognized as an effective and safe method for diagnosing brain tumors and became the gold standard for diagnosis. In a metaanalysis published in 1998, Hall et al. reported 0.7% mortality and 3.5% morbidity rates after stereotactic biopsy of intracranial lesions [9]. However, more recent United States registry data suggest that the death rate is approximately 3% with this procedure [14, 20]. In studies containing over 100 patients, endoscopic biopsy was associated with lower mortality rates, in the range of 0.0–0.3%, and complication rates of 11.2–12.9% [5, 12]. By contrast, a metaanalysis by Somji et al. [24], published in 2015, revealed a mortality rate of 2.2% and a major morbidity rate of 3.1% with endoscopic biopsy.

Despite the aforementioned data, no reliable published study has directly compared outcomes associated with the two biopsy methods or described the results of stereotactic biopsies of intracranial tumors involving ventricles. We therefore conducted a study to compare outcomes, including diagnostic effectiveness and complication rates, between endoscopic and stereotactic biopsies of lesions in this area.

## Methods

### Patients

#### Inclusion criteria

Data from all 1581 adults undergoing brain tumor biopsy at our institution between 2007 and 2018 were assessed for possible inclusion in this retrospective analysis. Of these, 211 patients had intracranial tumors involving the ventricles. A team of neurosurgeons experienced in stereotactic and endoscopic methods assessed their pretreatment imaging data and selected 119 patients with intraventricular or paraventricular tumors considered suitable for either stereotactic or endoscopic biopsy. In patients with multiple similar tumors, at least one tumor must have been suitable for stereotactic biopsy, and at least one tumor must have been suitable for endoscopic biopsy.

Tumors were considered intraventricular if the stereotactic biopsy trajectory passed through the ventricle (e.g., tumors of the septum pellucidum or foramen of Monro). Conversely, tumors were considered paraventricular if the stereotactic biopsy trajectory did not pass through the ventricle (e.g., thalamic tumors).

#### Exclusion criteria

The following exclusion criteria were applied: tumor not associated with the ventricular system; tumor not suitable for both endoscopic and stereotactic biopsies, in patients with a single tumor; lack of at least one tumor suitable for each type of biopsy, in patients with multiple similar tumors; and tumors penetrating into the ventricle, but for which performed or potential endoscopic pathway did not pass through the ventricle (e.g., skull base tumors for transsphenoidal biopsy and some cystic tumors near the ventricles).

### Surgical technique

The type of biopsy approach was based on the decision of the neurosurgical team. Imaging tests, personal preferences, and medical history (e.g., previous hydrocephalus treatment) affected the decision. For surgical planning, all patients were evaluated with magnetic resonance imaging (MRI). Stereotactic biopsies were usually performed under local anesthesia using a 4-pin stereotactic frame and BrainLab software with automatic CT/MRI image fusion. Microforceps were used to obtain serial biopsy specimens. When other procedures were performed simultaneously with the biopsy, general anesthesia was used if necessary. Endoscopic biopsies were performed under general anesthesia using a 30° endoscope (Minop, Aesculap). One or several samples of the tumor were obtained using forceps. Additional procedures, such as ventriculostomy or tumor cyst aspiration, were performed as needed. If the trajectories were significantly different, two separate burr holes were used for biopsy and ventriculostomy.

### Histopathology

In most cases, a neuropathologist present in the operating room confirmed the presence of pathological tissue by smear preparation. The final histologic diagnosis was based on the World Health Organization classification system. Some patients underwent subsequent open surgery to remove the tumor. In these cases, the histologic diagnosis based on biopsy specimens was compared with the diagnosis based on the resection specimen.

### Follow-up

Each patient underwent a postoperative CT scan within 24 h after surgery. They were then followed up on a regular basis, and their outcomes during the first 3 months postoperatively were analyzed.

### Statistical analysis

Diagnostic rates and complication rates were compared between the two biopsy methods. Fisher’s exact test was used to compare categorical factors. All *p* values were two-tailed, and the significance level was set at 0.05.

### Ethics considerations

Written informed consent was obtained for surgery from all participants. Ethical approval and individual consent to participate in the study were waived by the Ethics Committee according to local regulations and the tenets of the Helsinki Declaration because of the retrospective nature of this study that had no impact on the routine care.

## Results

### Clinical features

A total of 119 patients were included in the study. Their mean age was 47 years (range, 18–84 years). Detailed data regarding demographics, location of the biopsied lesion (intraventricular or paraventricular), and presence and severity of hydrocephalus are summarized in Table 1. A total of 120 tumors were considered suitable for biopsy, and 123 biopsies were performed: 85 stereotactic and 38 endoscopic. Four patients underwent both stereotactic and endoscopic biopsies. Two of these patients underwent stereotactic biopsy after nondiagnostic endoscopic biopsy. One patient had two different tumors in separate locations at different times (endoscopic biopsy in 2012, stereotactic biopsy in 2014). Another patient underwent simultaneous stereotactic biopsy and endoscopic third ventriculostomy (ETV), and because the tumor was clearly visible through the endoscope, samples were also obtained endoscopically. In 39 cases, additional procedures were performed at the same time as the biopsy (Table 2).

**Table 1:** Patient characteristics (*N* = 119)

Characteristics	Total	Stereotactic	Endoscopic
Number of biopsies	123	85	38
Males/females	57/62	42/43	17/21
Age, years	47 (18–84)	48 (18–78)	45 (18–84)
Paraventricular tumor	95	76	22
Intraventricular tumor	25	9	16
Hydrocephalus at initial diagnosis			
Significant hydrocephalus	73	45	32
Mild enlargement of ventricles or part of a ventricle	27	24	3
No ventricular dilation	19	16	3

Data are number or mean (range)

**Table 2:** Additional intraoperative procedures

Procedures	Total	Stereotactic	Endoscopic
All additional procedures	39	5	35
Endoscopic third ventriculostomy	26	2	25
Tumor cyst aspiration	10	2	8
Ventriculoperitoneal shunt	1	0	1
Septum pellucidotomy	1	0	1
Rickham reservoir placement	1	0	1

### Tumor pathology

The histopathologic diagnoses are presented in Table 3. In 5 cases, the biopsy samples were designated as nondiagnostic (2 stereotactic, 3 endoscopic). Overall, the diagnostic yield was 98% for stereotactic biopsies and 92% for endoscopic biopsies. Eleven patients underwent subsequent surgical resection of their tumor. In 7 cases (4 stereotactic, 3 endoscopic), pathologic examination of the resection specimen confirmed the initial biopsy diagnosis, but in 4 cases (3 stereotactic, 1 endoscopic), there were discrepancies between the diagnoses. When the numbers of nondiagnostic samples and incorrect diagnoses were added together, the total nondiagnostic biopsy rate was 6% (5 cases) for stereotactic biopsies and 11% (4 cases) for endoscopic biopsies. These rates were not significantly different between groups (Table 4).

**Table 3:** Distribution of pathological diagnoses

Histologic diagnoses	Total	Stereotactic	Endoscopic
Glioblastoma	25	23	2
Anaplastic astrocytoma III	21	16	5
Glioma malignum, nonspecified	2	2	0
Astrocytoma II	8	6	2
Astrocytoma pilocyticum I	1	0	1
Oligodendroglioma II	1	1	0
Oligoastrocytoma II	1	1	0
Ganglioglioma I	1	0	1
Anaplastic ependymoma III	5	4	1
Ependymoma II	10	7	3
Lymphoma	16	14	2
Metastasis	7	4	3
Craniopharyngioma	2	0	2
Germinoma	2	0	2
Pineoblastoma III	1	1	0
Pineocytoma I	1	0	1
Papillary tumor of pineal region III	1	0	1
Medulloblastoma	1	1	0
Colloid cyst	4	1	3
Glial cyst	3	0	3
Toxoplasmosis	1	1	0
Inflammation, nonspecified	1	0	1
Brain infarct	1	1	0
No significant pathology	2	0	2
Nondiagnostic samples	5	2	3

**Table 4:** Analysis of nondiagnostic biopsies

Histologic results	Total	Stereotactic	Endoscopic	*P* value
Nondiagnostic samples	5	2	3	–
Misdiagnosis	4	3	1	–
Total nondiagnostic biopsies	9 (7%)	5 (6%)	4 (11%)	0.456

### Complications

Complications occurred in 14 patients (11%): 10 (12%) in the stereotactic group and 4 (11%) in the endoscopic group (Table 5). Three bleeding episodes occurred within the first 24 h after biopsy. Two cases of delayed bleeding leading to death occurred within 1 month after biopsy of intraventricular tumors: 1 after stereotactic biopsy of a metastatic tumor and 1 after endoscopic biopsy of an ependymoma II. One patient developed severe, increasing brain edema with a fatal outcome on the third day after stereotactic biopsy of a larger, inoperable thalamic tumor (glioblastoma). Except for the complications resulting in death, all complications resolved with no permanent sequelae. There were no statistically significant differences in rates of complications or death between stereotactic and endoscopic groups.

**Table 5:** Postoperative complications

Complications	Total (out of 123)	Stereotactic (out of 85)	Endoscopic (out of 38)	*P* value
Early (< 24 h) postoperative intraventricular hemorrhage, resolved spontaneously	1	1	0	1
Early (< 24 h) epidural hematoma in the area of biopsy trajectory, required surgery	1	1	0	1
Early (< 24 h) intracerebral hematoma outside the biopsy area (in the cerebellum), resolved spontaneously	1	0	1	0.309
Delayed (> 48 h) intraventricular hemorrhage, resulting in death	2	1	1	0.524
Total significant bleeding (early or delayed)	5	3	2	0.644
Brain swelling, resulting in death	1	1	0	1
New seizures	3	3	0	0.552
Transient confusion and memory deficit (< 7 days)	2	1	1	0.524
Transient mild hemiparesis	1	1	0	1
Superficial wound infection	1	1	0	1
Urinary infection	1	0	1	0.309
Total morbidity	14	10	4	1
Total mortality	3	2	1	1
Hydrocephalus required treatment within 1 month after biopsy	6	6	0	0.176

During follow-up, 6 patients required additional surgery for hydrocephalus because of disease progression. All 6 of these patients had undergone stereotactic biopsy, although the rate of additional complications was not significantly different between groups (Table 5). There were no complications after the additional operations or other adverse consequences during the observation period.

The overall mortality rate was 2.4% for the 119 patients with lesions in the ventricular area. This was significantly higher than the 0.3% mortality rate for all 1581 patients undergoing biopsy of intracranial lesions at any location during the study period (*p* = 0.016; Table 6).

**Table 6:** Mortality rates after biopsy of intracranial lesions in the ventricular area and intracranial lesions in all locations

Biopsies and deaths	All intracranial biopsies	Intra- and paraventricular biopsies	*P* value
Number of biopsies	1581	123	–
All biopsy-related deaths	5 (0.3%)	3 (2.4%)	0.016

## Discussion

### Literature review

An intensive PubMed search revealed that in recent publications, reports of endoscopic methods have dominated the topic of biopsies for pathology in the region of the ventricular system. Many of these reports, including the largest multicenter study [5], mention numerous advantages of endoscopic biopsies over stereotactic biopsies, especially for intraventricular tumors. The literature therefore suggests that endoscopic biopsies are better options than stereotactic biopsies, and a few of us also prefer the endoscopic technique. However, these preferences are based primarily on intuition, as no published study has confirmed the advantages of one method over the other for tumors involving the ventricular system.

Stereotactic and endoscopic biopsies have been compared in large groups of patients only for pineal tumors. Somewhat unexpectedly, the studies by Balossier et al. [3, 4] demonstrated slightly superior effectiveness and safety with stereotactic biopsies for pineal tumors. Kinfe et al. [16] compared biopsy approaches for only paraventricular tumors, but their study was confounded by selection bias (e.g., the stereotactic group included cases considered unsuitable for endoscopic biopsy). Therefore, conclusions from that study are of limited value. Although many papers reported the results of intracranial stereotactic biopsies, including papers involving very large groups (i.e., thousands) of patients [9, 14, 20, 22], these publications either did not report the results for tumors involving the ventricles or included very small numbers of patients with tumors in this region. We identified only a few papers containing more than 10 cases of para- and intraventricular lesions [1, 2, 7, 8, 10, 16, 27]. Livermore et al. [18]. reported only that there was a significant increase in hemorrhage rate with biopsies of deep versus superficial intracranial tumors. Because of the small amount of data and inconsistency between study results, no metaanalysis has been performed comparing biopsy methods for intracranial tumors involving the ventricles.

### Case selection

In this study, we included only cases considered suitable for either type of biopsy at the time of the initial diagnosis. The type of biopsy actually performed was not randomized but depended on the neurosurgeon’s individual decision. Selection bias remains a possibility in our study. After their initial diagnosis, some patients included in the study were effectively treated for hydrocephalus at other hospitals before being referring to our institution for biopsy, and at that time, endoscopic biopsy was no longer recommended. However, if the tumor had been considered suitable for both biopsies at the time of the original diagnosis, then the patient was included in the study. Because of this, there were more stereotactic than endoscopic biopsies in the current study.

An original objective of this study was to perform separate comparisons for intraventricular and paraventricular tumors. However, the number of cases in the intraventricular group was small, prohibiting separate evaluations.

### Histopathology

In 2 cases, the histopathologic diagnosis was “no significant pathology”. It was uncertain whether these were diagnostic or nondiagnostic biopsies; nevertheless, the tissue samples were adequate for histopathologic evaluation, control imaging studies confirmed the site of collection within the radiologic lesion, and the lesions did not change during further follow-up. Therefore, we considered the diagnosis to be accurate and classified these cases as diagnostic.

In addition to histopathologic examination, molecular studies have been recently introduced for tumor assessment. However, results of these studies were available for only some of the tumors evaluated in this study, so we did not include these findings in our analyses.

### Complications

The 2 patients with delayed intraventricular bleeding displayed very similar postoperative courses. Their CT scans on the day after biopsy showed only a small amount of blood in the ventricles, with no neurologic sequelae. This bleeding was therefore considered nonserious. However, the neurologic status of both patients began to deteriorate more than 2 days after the biopsy, and subsequent CT examinations showed gradually increasing blood volume in the ventricles. Extraventricular drains were inserted, but despite this treatment, the neurologic status of both patients continued to worsen, leading to death within a month. These deaths could possibly have been prevented with more aggressive treatment, such as resection of the bleeding tumor.

### Additional procedures

The advantages and disadvantages of both biopsy methods are well known. Perhaps the most important advantage of the endoscopic method is the ability to perform other procedures during the same operation as the biopsy. In our study, hydrocephalus treatment and tumor cyst aspiration were performed more frequently with endoscopic biopsies, resulting in a reduction of intracranial mass effects. Thus, endoscopic biopsies were often therapeutic, as well as diagnostic. If additional procedures were not performed, the risk of endoscopic biopsies themselves may have been lower. On the other hand, however, for stereotactic biopsies, the cumulative risk of ventriculoperitoneal shunt placement and biopsies was not higher than the risks associated with the biopsies themselves, as we did not observe any shunt-related complications in the stereotactic biopsy group. It is possible that the main benefit of combining procedures is the shorter duration of entire treatment. Especially in cases of hydrocephalus occurring within a month of biopsy, oncologic treatment was unnecessarily delayed. In at least some of these cases, ETV could have been performed prophylactically if endoscopic biopsy had been used. Nonetheless, it would be difficult to design a study evaluating the effects of biopsy method on treatment duration because of the many confounding variables that would be involved.

### Limitations

The limitations of the study are its retrospective character, selection bias due to the choice of the biopsy type by the surgeon, evaluation of the results from only one center, and only one of the different types of stereotactic biopsies was used. In our department, we routinely use microforceps for stereotactic biopsies. We used side-cutting needle and spiral needle in only a few specific cases in other areas. In the ventricular area we prefer to use microforceps because the side-cutting needle requires a longer trajectory which increases the risk of puncture the ependyma. There is not enough evidence to conclude that one tool is significantly better than the other. Nevertheless, we cannot exclude that the choice of biopsy method affects the results. Kreth et al. in 2001 [17] published a very low complication rate and very high diagnostic rate using microforceps. On the other hand the use of a side-cutting needle has been reported more frequently in recent years, but this trend is not supported by scientific evidence, but only by some opinions [21]. We can only find a comparison of tissue collection methods in a very small study of AIDS patients [13] and of dead pig brains [25]. In meta-analyzes [9, 11, 15] and systemic review [22] the results were not compared depending on the method of tissue collection during stereotactic biopsies. The neurosurgeons participating in this study usually perform frame-based biopsies, instead of navigational, frameless biopsies. Consequently, only frame-based cases were included in the stereotactic biopsy group. However, the results are likely generalizable to all types of stereotactic biopsies, as previous studies confirmed that effectiveness and safety are not significantly different between stereotactic frameless and frame-based needle biopsies [6, 19, 23, 26].

## Conclusions

The mortality rate was higher after biopsies of intracranial tumors involving the ventricles than after biopsies of all intracranial tumors (in any location). The rates of total nondiagnostic biopsies and complications after endoscopic and stereotactic brain biopsies in the ventricle area were similar. The only possible, but not fully confirmed, advantage of endoscopic biopsies over stereotactic biopsies is the ability to simultaneously treat hydrocephalus and thereby shorten the time for determining the pathologic diagnosis and treating hydrocephalus.

## Data Availability

All collected data can be made available on a reasonable request.
