# Correction to: Endoscopic versus stereotactic biopsies of intracranial lesions involving the ventricles

**DOI:** 10.1007/s10143-021-01497-2

**Published:** 2021-02-23

**Authors:** Marcin Birski, Jacek Furtak, Kamil Krystkiewicz, Julita Birska, Karolina Zielinska, Paweł Sokal, Marcin Rusinek, Dariusz Paczkowski, Lukasz Szylberg, Marek Harat

**Affiliations:** 1 Neurosurgery Department, 10th Military Research Hospital, ul. Powstancow Warszawy 5, 85-681 Bydgoszcz, Poland; 2 Department of Neurosurgery and Neurology, Jan Biziel University Hospital Collegium Medicum Nicolaus Copernicus University, Bydgoszcz, Poland; 3 Department of Clinical Pathomorphology, Collegium Medicum Nicolaus Copernicus University, Bydgoszcz, Poland; 4 Department of Tumor Pathology and Pathomorphology, Oncology Center, Bydgoszcz, Poland; 5 Department of Pathomorphology, 10th Military Research Hospital, Bydgoszcz, Poland

**Correction to: Neurosurg Rev**

**https://doi.org/10.1007/s10143-020-01371-7**

The article Endoscopic versus stereotactic biopsies of intracranial lesions involving the ventricles, written by Marcin Birski, Jacek Furtak, Kamil Krystkiewicz, Julita Birska, Karolina Zielinska, Paweł Sokal, Marcin Rusinek, Dariusz Paczkowski, Lukasz Szylberg, and Marek Harat, was originally published electronically on the publisher’s internet portal on August 17, 2020 without open access. With the author(s)’ decision to opt for Open Choice the copyright of the article changed on September 15 2020 to © The Author(s) [2020] and the article is forthwith distributed under a Creative Commons Attribution 4.0 International License, which permits use, sharing, adaptation, distribution and reproduction in any medium or format, as long as you give appropriate credit to the original author(s) and the source, provide a link to the Creative Commons licence, and indicate if changes were made. The images or other third party material in this article are included in the article's Creative Commons licence, unless indicated otherwise in a credit line to the material. If material is not included in the article's Creative Commons licence and your intended use is not permitted by statutory regulation or exceeds the permitted use, you will need to obtain permission directly from the copyright holder. To view a copy of this licence, visit http://creativecommons.org/licenses/by/4.0/.

The original article has been corrected.

